# Climate change, large fires, and cultural landscapes in the mediterranean basin: An analysis in southern Spain

**DOI:** 10.1016/j.heliyon.2023.e16941

**Published:** 2023-06-02

**Authors:** M. Moreno, C. Bertolín, D. Arlanzón, P. Ortiz, R. Ortiz

**Affiliations:** a Dpt. Physical, Chemical and Natural Systems, University Pablo de Olavide, Seville, Spain. ES-41013; b Dpt. of Mechanical and Industrial Engineering, Norwegian University of Science and Technology, Trondheim, Norway

**Keywords:** Traditional landscapes, Satellite resources, Fire risk, Andalusia, CHIRPS, MODIS

## Abstract

Understanding the factors that influence fire regimes in Mediterranean climates is essential to reduce their risk. This research uses Climate Hazards Group InfraRed Precipitation with Station (CHIRPS) and Moderate-Resolution Imaging Spectroradiometer (MODIS) satellite resources to evaluate recent changes in land surface temperature, precipitation, and vegetation and their effects in the occurrence of large fires in the Mediterranean Basin. The results of the analysis of 335 fire events occurred in southern Spain from 2001 to 2020 show an increase in hazardous meteorological factors linked to droughts and thermal anomalies. The study also examines the potential of preserving traditional landscapes to minimize such risk. In fact, the maintenance and recovering of traditional agro-pastoral activities is an effective option to reduce flammability and increase the resilience of cultural landscapes in hazardous climatic conditions.

## Graphical abstract

**Figure:**
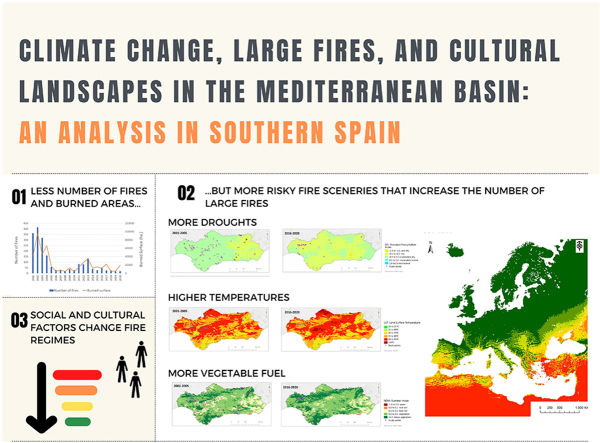

## 1 Introduction and research aims

Fires are natural phenomena that historically have been part of the ecosystem in Mediterranean and semi-arid climates, and have influenced their genetic diversity and variability [[1], [2], [3], [4], [5]]. Despite this, the impact of large fires is currently increasing in the Mediterranean basin, that is becoming one of the areas with the highest risk of fires in the world [[6], [7], [8]].

The magnitude and duration of large fires can favour soil erosion [9,10], modify the micro-climate [11], and have serious impacts on ecosystem services and the inhabitants of the affected areas [12]. In recent years, the occurrence of firestorms, i.e., fires capable of generating pyro cumulus clouds that modify local meteorology [13], has increased with devastating effects [[14], [15], [16]].

Regarding weather conditions, the probability of a fire becoming a large fire depends mainly on temperature, lack of precipitation, and wind. In the Mediterranean basin, climate change has caused an increase in temperature and intense droughts with a consequent higher probability of occurrence of large fires [[17], [18], [19], [20]].

Nowadays, there are different methods or indices to monitor the meteorological hazard of fire [21,22]. Examples of these are the Keetch-Byram Drought Index (KBDI) [23], Mark 3 and Mark 5 fire danger index (FFDI) [24,25], or the National Fire Danger Rating System (NFDRS) [26]. The most used in Mediterranean Basin is the Fire Weather Index (FWI) [[27], [28], [29], [30], [31]]. It is a daily meteorology index based on dry air temperature (°C), relative humidity (%), wind speed (km/h), and rainfall over the last 24 h (mm) [29]. Although the meteorological data for its calculation are usually obtained from ground meteorological stations, satellite products have recently been used to calculate it [32]. In Europe, the GWIS viewer (https://gwis.jrc.ec.europa.eu/) enables online access to these products [33] as well as to forecasts up to the year 2098. These are derived from climate models such as CNRM-CM5, HadGEM2-ES, or MPI-ESM-LR based on projected greenhouse gas concentration scenarios as reported by the Intergovernmental Panel on Climate Change [32].

Besides the weather factors, the risk also depends on fuel characteristics on land: how much fuel is available, how flammable, and how combustible it is. In Mediterranean climates, during the summer, the lack of water reduces vegetation's photosynthetic activity and increases flammability [10].

Vegetation indices based on reflectance levels in the Near-infrared (NIR) and shortwave infrared (SWIR) have been used to measure the fuel availability. Examples of these are the Normalized Difference Infrared Index (NDII), the Vegetation Condition Index (VCI) [34,35] and the Normalized Difference Vegetation Index (NDVI) that assess vegetative density, vegetation susceptibility, and changes in photosynthetic activity [36,37]. On smaller spatial scales, the use of LIDAR images has also been used to determine the density of vegetation cover [[38], [39], [40]].

Typically, an increase in biomass means a greater availability of vegetative fuel in the summer, resulting in higher fires intensity [10]. Although generally a positive trend between the accumulation of dead biomass and the time elapsed since the last fire may be detected, in Mediterranean vegetation, such rule does not apply. As a matter of fact, in the Mediterranean Basin, the types of vegetation that appear immediately after a fire are more flammable and accumulate more dead biomass than the species that appear at later stages [41]. In addition, the complex relationship between climatic-vegetative variations and fires occurrence poses difficulties in developing predictive models of fire risk. In fact, droughts in the short term reduce the humidity of the vegetal cover and increase fire risk, while in the long term, they reduce the available biomass, contributing to a decrease in fire risk. On the other hand, rainfalls have the opposite effect. If they occur just before the fire season, they reduce the imminent risk of fire, while if they happen abundantly in previous years, they increase biomass availability, thus strengthening the intensity of possible fires [42].

Besides, although the intensity and spread of fires are strongly conditioned by meteorological and vegetative conditions, most fires have anthropic causes closely related to the way of inhabiting a territory [10,19,43,44]. The abrupt changes that cultural landscapes have undergone throughout the 20th and 21st centuries have contributed to modify fire regimes resulting in higher impacts [43,45].

Currently, there are different models to assess fire risk that include socio-demographic variables such as the density of crops or the proximity to roads [42,44,46,47]. Despite this, there is no consensus regarding which variables must be included, and how they influence the occurrence of fires.

**Fig. 1:**
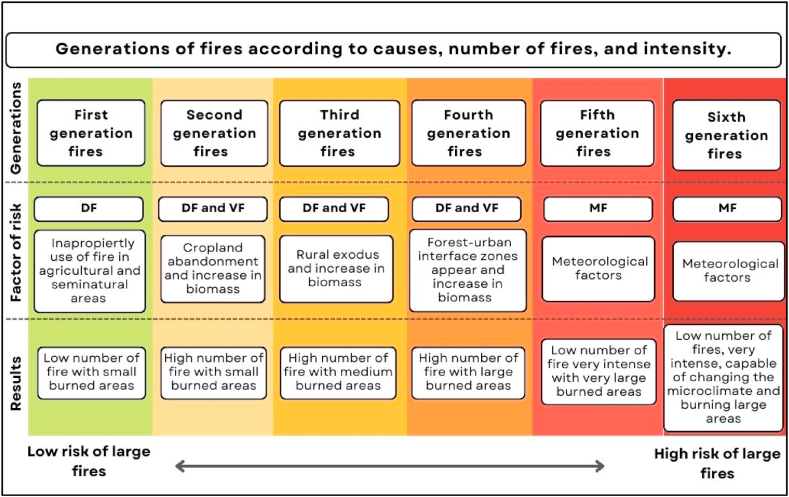
Simplified representation of the natural and anthropogenic factors that affect the risk of fire according to the classification in fire generations established by Costa et al. [48].

An example of a simple explanatory model that takes into account climatic, vegetative, and social factors to evaluate fire risk is the *Fire Generations model* [48]. Fig. 1 shows the six socio-climatic scenarios that, according to this model, condition fire regimes. *First generation fires* would be associated with rural use of the countryside; *second generation fires* are related to the abandonment of agricultural land and the growth of scrub; *third generation fires* would be caused by rural exodus and increase of biomass; *fourth generation fires would be caused* by the accumulation of vegetation in forest areas and the increase of the inhabited natural interfaces, and the *fifth and sixth generation fires* to the effects of climate change. In general, *the first and second generation* scenarios cause a high number of low-intensity fires, while *the third to sixth generation scenarios* present a lower frequency of fires but of greater intensity, with more burned surfaces and difficult to control [48]. Thus, the risk of large fires increases in higher fires generations.

Models that consider both social and climatic factors are useful management tools in the current context of climatic change. Despite the increase of climate hazards, they allow to disclose what could be effective demo-social policies for reducing the fire hazard and enhancing the resilience of local communities. By adopting these assessment models, sustainable management practices of cultural landscape could be encouraged.

This study analyzes what is the impact of combined meteorological, vegetative, and social factors in increasing the risk of fire in Southern Spain. It demonstrates how crucial is becoming, in the context of climate change, developing effective fire risk management plans. Therefore, the main objective of this research is to investigate the driving factors that are transforming the occurrence of fires in the Andalusia region selected here as a representative area with Mediterranean and semi-arid climates. To achieve this, three specific objectives have been proposed: 1) the evaluation of the annual changes in Land Surface Temperature (LST), precipitation, vegetative density, and population distribution over the 2001–2020 period using satellite resources and Geographic Information System (GIS); 2) the analysis of the annual change in the number of fires and burned hectares over the 2001–2020 period; 3) the analysis of the characteristics of the burned surfaces over the 2001–2020 period from the study of the occurred 335 fires.

Adopting the fire generation model by Costa et al. [48], the datasets described above have been discussed at the light of the future climatic projections for the Mediterranean basin and the possible sociodemographic changes that may influence the occurrence of fires. The results provide an understanding of how meteorological, vegetative, and population factors contribute to generate high-risk scenarios in Andalusia. Furthermore, they offer relevant information for the development of models capable of managing multi-risk scenarios in Mediterranean landscapes.

## 2 Study region and fire analyzed

**Fig. 2:**
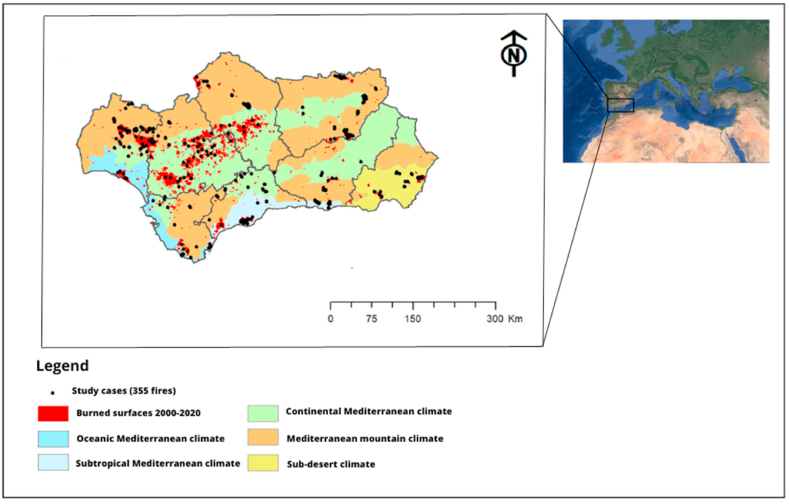
Climatic zones in Andalusia. Burned surfaces are highlighted in red, and study cases are marked with black dots. (For interpretation of the references to color in this figure legend, the reader is referred to the Web version of this article.)

The study area includes the political boundary of Andalusia, a region located to the southwest of the Mediterranean basin. According to the Köppen climate classification, Andalusia has a Mediterranean climate (Csa) with mild, humid winters and very hot summers with hardly any rainfall. The study area includes Oceanic Mediterranean climate, Continental Mediterranean climate, subtropical Mediterranean climate, sub-desert climate, and Mediterranean mountain climate (Fig. 2).

Natural and forest areas occupy 49% of the territory. 37% of them have mixed masses of trees and scrub and 10% masses of dense trees [49]. The coniferous forests are located to the eastern of Andalusia and the sclerophyll forests of oaks are in the western part [50]. Agricultural lands occupy 47% of the territory and are in the inland [49]. Urban areas occupy the remaining 2–4%.

The 335 fires analyzed over the recent two decades (Fig. 2, black dots) include two bioclimates (humid and arid Mediterranean climate) and seven different land covers (mixed forest, coniferous forest, broad-leaved forest, scrub, transition scrub, grassland, and crop fields).

Regarding relief, Andalusia largely presents flat surfaces and in more than 41% of the territory, the slopes lower than 10% [49].

Typically, winds are dry and warm during the summer months. However, there are different types of winds that vary based on their direction, speed, and humidity content. Winds blowing from the south carry dust and sand particles in suspension, while those from the west can produce gusts of up to 100 km/h [51].

Currently, Andalusia has a population of 8, 464,411 and a mean density of 97 people per hectare. The distribution within the region varies significantly, with provincial capitals being the most populous areas [51].

Until the mid-20th century, the economy of Andalusia depended primarily on agriculture and livestock, but since 1960, there has been an increase in activities related to the services and tourism sectors. This change has been accompanied by a rural exodus and strong urbanization of large and coastal cities. Nowadays, the economy is mainly based on tourism and agriculture. The cultural heritage, together with natural landscapes and beaches, are the main attractions according to tourists [51,52].

## 3 Materials and methods

### 3.1 Materials

The values of LST, precipitation, vegetative density and burned surfaces in Andalusia between 2001 and 2020 were obtained through the statistical analysis of historical series of satellite resources in Google Earth Engine (GEE) (https://earthengine.google.com/).

The burned surfaces have been classified and mapped according to year and season (spring, summer, autumn, and winter) from the combined product MCD64A1.006 from the satellites Terra and Aqua. This satellites product has a spatial resolution of 500 m and a temporal resolution of 1 month indicating burned surfaces from a burn-sensitive vegetation index obtained from the Moderate-Resolution Imaging Spectroradiometer (MODIS) surface reflectance bands 5 and 7 (https://lpdaac.usgs.gov/products/mcd64a1v061/). To avoid deformation errors caused by the projection system, the images have been vectorized before quantifying the burned hectares.

The land cover of the burned surfaces was obtained from Corine Land Cover. This is a geospatial resource that uses satellite data to create an inventory of land cover types in Europe (https://land.copernicus.eu/pan-european/corine-land-cover/clc2018).

LST data were obtained from the Advanced Spaceborne Thermal Emission and Reflection Radiometer (ASTER) and the Moderate-Resolution Imaging Spectroradiometer (MODIS) aboard the Terra satellite. Of all the MODIS products for LST and emissivity, the MOD11A1.006 resource (https://lpdaac.usgs.gov/products/mod11a1v006/) was employed, which is a product derived from MOD11_L2 (Land Surface Temperature/Emissivity Daily 5-Min L2 Swath 1 km). The MOD11 products provide estimates of the land cover diurnal and nocturnal temperature and emissivity. The algorithm used estimates temperature and land cover from satellite images in the Infrared bands. It employs the split-window LST algorithm (Wan and Dozier, 1996) to calculate temperature based on the linear difference between the brightness temperature of bands 31 and 32 and land cover types. The temperature is indicated in degrees Kelvin. MOD11A1.006 has a spatial resolution of 1 km and a temporal resolution of 1 day. The use of this resource has been widely validated by other researchers with in-situ measurements in temperature ranges from −10 °C to 58 °C and column water vapor ranging between of 0.4–4.0 cm [53,54].

Precipitation data were obtained from the Climate Hazards Group InfraRed Precipitation with Station data (CHIRPS) (https://www.chc.ucsb.edu/data/chirps). CHIRPS provides precipitation data since 1981 at 5 km spatial resolution and daily temporal resolution. The CHIRPS algorithm uses monthly precipitation climatology, satellite observations, precipitation estimates from NOAA, and observed data from terrestrial weather stations to calculate precipitation data [55,56]. Its use has been validated in numerous research studies [57,58].

Vegetative density was obtained from the Normalized Vegetation Index (NDVI) from the satellite resource MOD13A1.006 Terra Vegetation Indices. The NDVI is a standardized index utilized to quantify and assess the quantity and condition of vegetation coverage within a particular geographic region. Its methodology involves a mathematical comparison between the values of absorption and reflection of red and NIR light to understand how healthy vegetation is. This satellite product has a spatial resolution of 500 m and a temporal resolution of 15 days. The NDVI values come from National Oceanic and Atmospheric Administration-Advanced Very High-Resolution Radiometer (NOAA-AVHRR) (https://lpdaac.usgs.gov/products/mod13a1v006/).

Finally, FWI forecasts for Mediterranean basin have been prepared from the Copernicus cartography obtained from the ERA 5 reanalysis data [32] (https://cds.climate.copernicus.eu/cdsapp#!/dataset/sis-tourism-fire-danger-indicators?tab=overview).

Population density maps and population trends have been obtained from the Web Map Service WMS: Population and Demography Statistics of Andalusia, which records the number of inhabitants and annual rate of variation by municipality. This resource is available on the Andalusian Spatial Data Infrastructure (https://www.ideandalucia.es/catalogo/inspire/srv/spa/catalog.search#/metadata/ae809d89-336d-40b8-bed4-9db7c0422ddb).

### 3.2 Method

To analyze the effects of climate change at territorial scale, meteorological and vegetation satellite resources have been statistically analyzed using Javascript in GEE.

Annual and six months precipitation anomalies have been calculated by map algebra based on the Standardized Precipitation Index (SPI) (eq. (1)). The SPI is a measure used to quantify the deficit or excess of precipitation in a specific area over a certain time period capable to detect the variability of precipitation and its temporal distribution. It is based on observed precipitation values in an area and a reference period [59].

Mean, maximum, and minimum annual and seasonal LST have been calculated by temporal reducers. Seasonal anomalies of LST and NDVI have been calculated by subtracting from the mean of the last 20 years, the mean recorded for each of the analyzed years and seasons (eqs. (2), (3)).).

The mean values distributed in four periods of five years (2001–2005, 2006–2010, 2011–2015, and 2016–2020) were calculated using statistical reducers. Then, pixels above 45 °C, with an SPI < -0.1, and an NDVI >0.4 were counted for LST, drought, and vegetative density maps, respectively. The trend in territorial variables was obtained based on the changes identified in the number of pixels that recorded values above these thresholds in each of the four analyzed periods.

To evaluate changes in fire regimens during the last 20 years, the number of fires and burned hectares by year and season have been mapped in ArcGIS 10, a Geographical Information System from ESRI. The time series have been studied calculating the relative variation and the rate of the number of fires and burned hectares according to year and season. The rate is a relative measure that indicates the frequency with which something happens in a territory during a certain period. As an example, the rate of burned hectares by season was obtained dividing the burned hectares by season by the total burned hectares that year. Relative variation was obtained by subtracting from the burned hectares in the previous analyzed year, the burned hectares in the current year under analysis, and then dividing the difference by the total burned hectares during the entire analyzed period. Results are expressed as a percentage (eq. (4)).

To analyze the specific characteristics of the fires, discrete values of LST, precipitation, and vegetative density immediately before the fire started were obtained on 355 burned surfaces over the last 20 years. The construction of box plots and the comparison of the range, maximum, minimum, median, and quartiles of the collected values have allowed for the identification of trends and changes in each of the analyzed variables.

Finally, the risk associated with the identified changes has been discussed based on the climate forecasts for the Mediterranean basin and the social model proposed by Costa et al. [48].

(1) $$SPI=\frac{P\left(i\right)-P\left(ij\right)}{\sigma P\left(ij\right)}$$

Where SPI is the Standardized Precipitation Index, P(i) is the actual precipitation, P(ij) is the precipitation in a return period, and σP(ij) is a standard deviation in precipitation during a return period.

(2) TA=T(ij)−T(i)

Where TA is the LST anomaly, T(i) is the actual seasonal LST, and T(ij) is the seasonal LST during a return period.

(3) NDVIA=NDVI(ij)−NDVI(i)

Where NDVIA is the normalized difference vegetation index anomaly, NDVI*(i)* is the actual s*easonal normalized difference vegetation index, and NDVI (ij) is the seasonal normalized difference vegetation index in a return period.*

(4) $$RV=\frac{BSR-BSO}{BSO}\times 100$$

*Where* RV is the relative variation of burned hectares in %*, BSR is the most recent value of* burned hectares under analysis*, and BSO is the past value of* burned hectares*.*

## 4 Results

### 4.1 Changes of LST, precipitation, vegetative density, and population distribution in Andalusian territory

The satellite-derived territorial cartography presents the changes in LST, precipitation, and vegetative density that have occurred in Andalusia over the past 20 years.

**Fig. 3:**
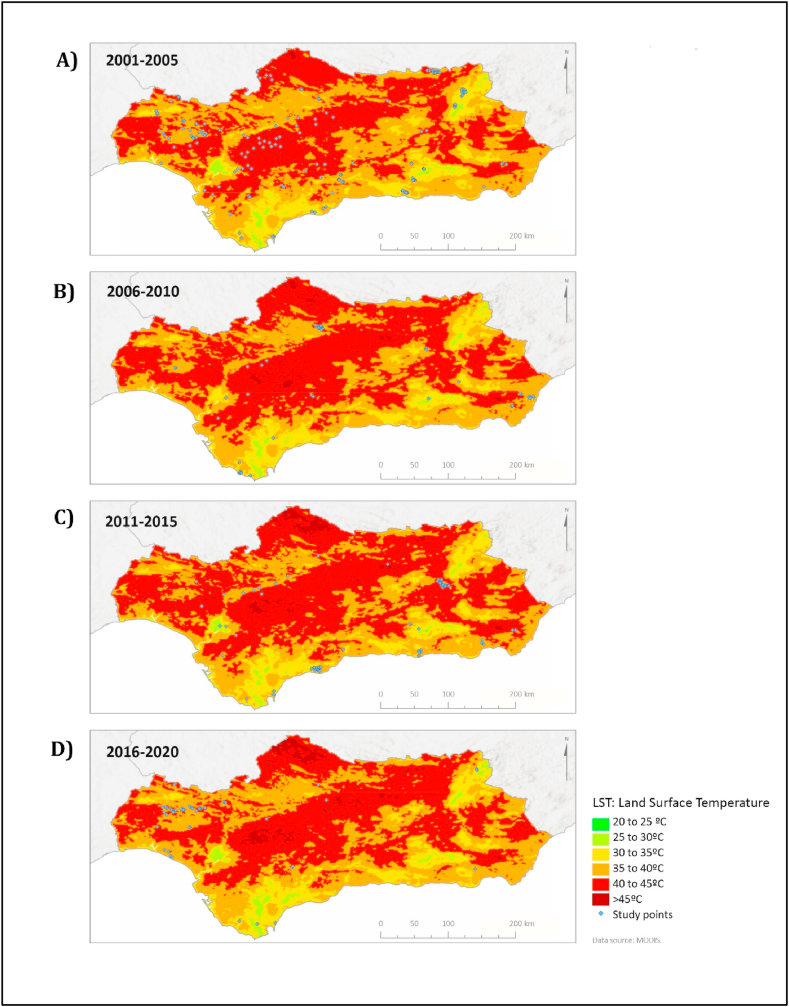
LST and studied fires marked in blue dots. Means of the values registered during consecutive 5-years summer periods from 2001 to 2020. A) 2001–2005 LST; B) 2006–2010 LST; C) 2011–2015 LST; D) 2016–2020. Maps show an increase in the surfaces that present more than 45 °C.Values obtained from the MODIS MOD11A1.006 satellite resource. (For interpretation of the references to color in this figure legend, the reader is referred to the Web version of this article.)

Fig. 3 shows the mean LST values, and the fire locations studied, marked in blue. The high LST observed in Andalusia indicates the presence of extensive areas at extreme risk of fire during summer and autumn. Agricultural areas located far from the coast are those where most fires occurred and are the most affected by high LST (>45 °C).

Furthermore, Fig. 3 depicts a rising trend that favors the expansion of areas affected by high LST during the fire season. Table 1 quantifies this trend by accounting for the number of pixels and their corresponding hectares (ha) affected by high LST. Specifically, during the summer seasons of 2001–2005 (Fig. 3A), the number of ha surpassing 45 °C was 14,233; during 2006–2010 (Fig. 3B), it was 98,281 ha; for the summers of 2011–2015 (Fig. 3C), it was 185,751 ha, and for the recent summer season of 2016–2020 (Fig. 3D), it amounted to 242,689 ha. These numbers corroborate the local impact of forecasts that predict gradual temperature increases attributed to climate change [[60], [61], [62]]. Despite this, this contribution did not find a direct relationship between the years with the highest number of fires-burned hectares, and the changes in inter-annual LST.

**Table 1:** Area (ha.) that presents high fire risk characteristics according to temperature, drought, and vegetative density for the 5-years periods analyzed in Andalusia.

Years	Summer LST >45 °C (ha)	Drought SPI < -0.1 (ha)	Vegetative density NDVI >0.4 (ha)
2001–2005	14,233	2,493,371	1,246,685
2006–2010	98,281	0	1,300,500
2011–2015	185,751	4,275, 243	1,448,785
2016–2020	242,689	6,363,400	1,554,466

**Fig. 4:**
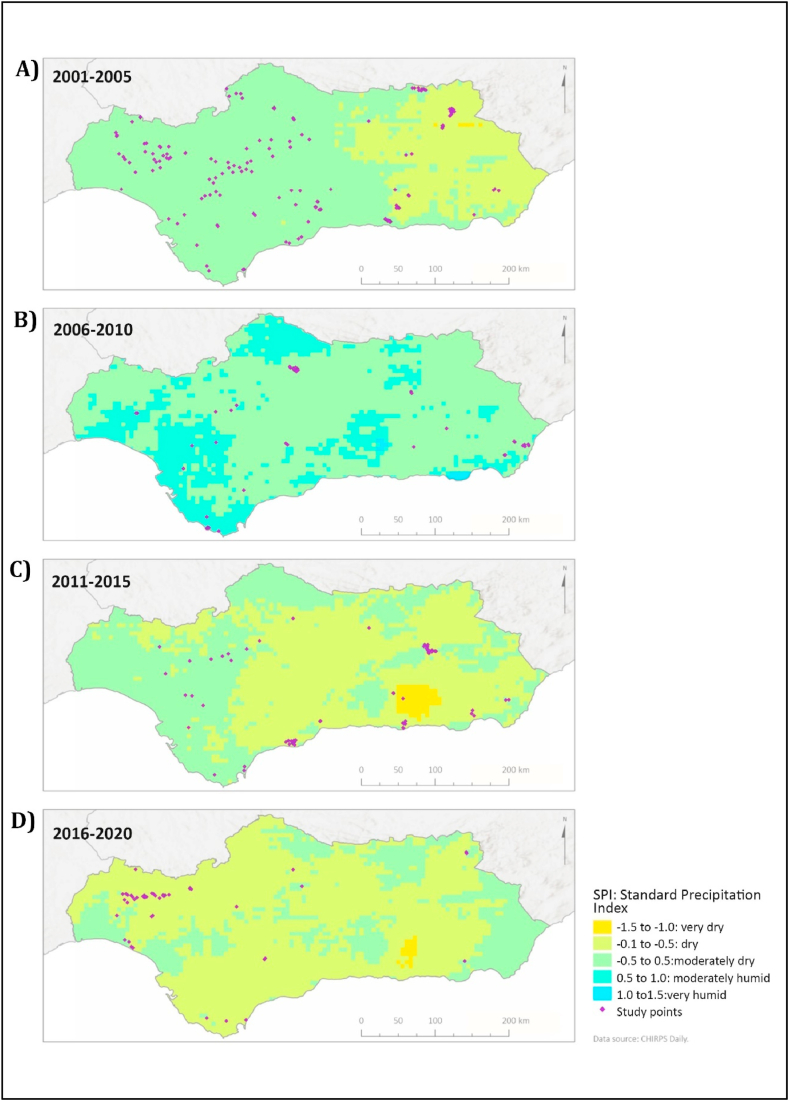
Annual Standard Precipitation Index and studied fire marked in purple dots. Means of the values registered during consecutive 5-year periods from 2001 to 2020. A) 2001–2005 LST; B) 2006–2010 LST; C) 2011–2015 LST; D) 2016–2020. Yellowish colors show the areas where it rains less than normal, and blueish colors show the areas where it rains more. Values obtained from CHIRPS satellite resource. (For interpretation of the references to color in this figure legend, the reader is referred to the Web version of this article.)

Regarding precipitation, the SPI values provide an indication of precipitation anomalies and droughts at annual (12 months) and semi-annual (6 months) resolution. Fig. 4 shows a tendency for fires to occur in moderately dry or very dry areas (yellow or light green color in Fig. 4). Besides, it shows greater occurrence of droughts since 2011 (Fig. 4C), especially during the second half of the year, which includes the summer and autumn months. So, the number of ha with SPI less than −0.1 between 2001 and 2005 (Fig. 4A) were 2,493,371, while between 2016 and 2020 (Fig. 4 D) this number increased to 5,363,400 ha. In general, the most affected territories are in the Southeast, an area with a sub-desert climate. Nonetheless, the interannual variation of SPI is very large and affects the territory differently. For instance, between 2006 and 2010 (Fig. 4B), a significant decrease in the areas affected by droughts was observed (Table 1), indicating that the differences in SPI are related to the influence of teleconnection patterns such as WeMO and NAO [63,64].

**Fig. 5:**
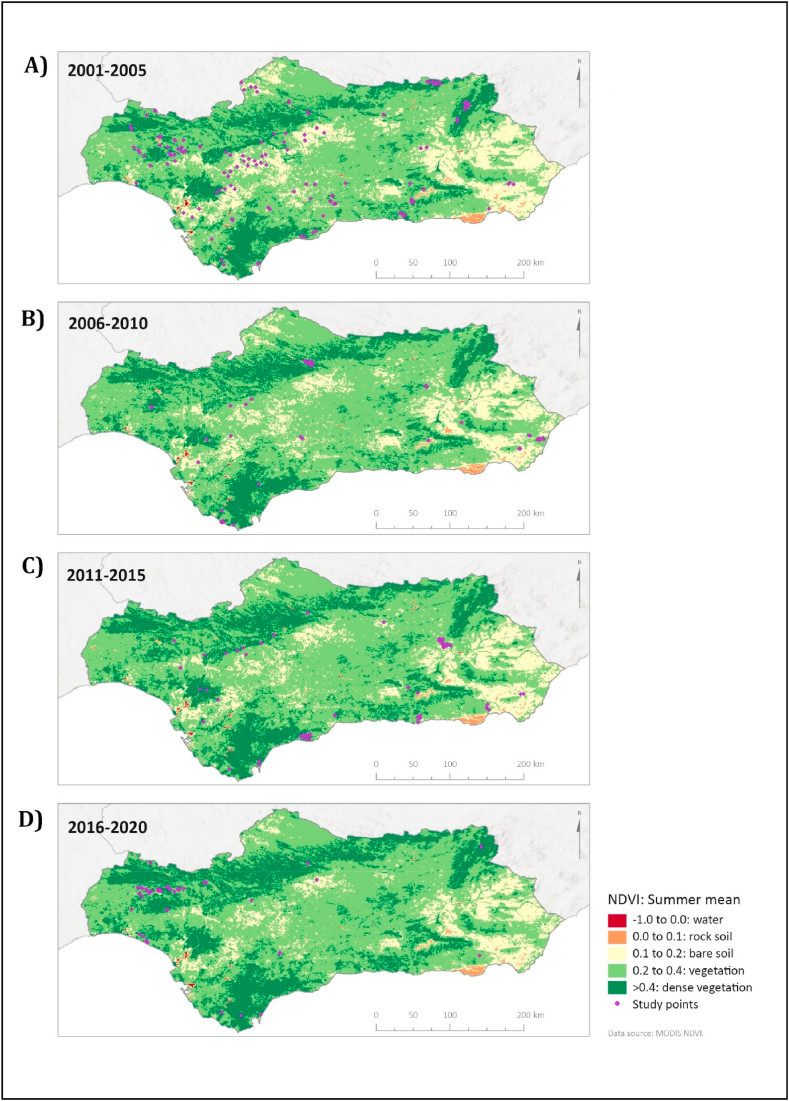
NDVI. Means of the values registered during the summer in subsequent periods of 5 years. A) 2001–2005 LST; B) 2006–2010 LST; C) 2011–2015 LST; D) 2016–2020. Maps show an increase in dense vegetation (>0.4 NDVI). Values obtained from MODIS Terra Vegetation Indices: MOD13Q1.006 satellite resource.

Fig. 5 shows the vegetative density according to NDVI levels, which show notable increase in recent years. Specifically, Table 1 shows 1,246,685 ha with dense vegetation (NDVI>0.4) between 2001 and 2005 (Fig. 5A), while between 2016 and 2020 (Fig. 4D), this number increased to 1,554,466 ha of dense vegetation. The North forests and inland agricultural areas are the regions that have recorded the most significant vegetative development over the last two decades.

**Fig. 6:**
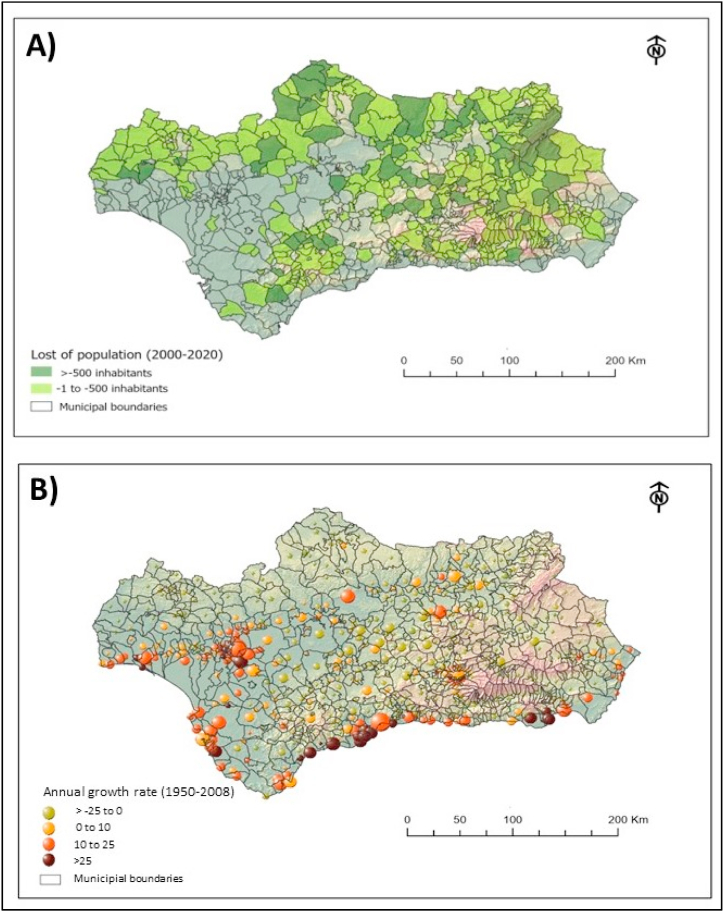
Change in demography in Andalusia: A) Lost of population between 2000 and 2020; B) Population growth rate between 1905 and 2008.

From a demographic perspective, a positive increase in population density has been observed in Andalusia during the analyzed period. In 2000, there were 7,340,052 inhabitants, whereas in 2020 the population has increased to 8,464,411 [65]. Fig. 6A shows in dark green and light green the places that, despite the overall increase, have lost population between 2000 and 2020. Fig. 6B shows the rate of population variation recorded between 1950 and 2008, enabling the identification of long-term general trends. Green circles indicate places where the population has decreased, while orange and red indicate an increase. The size of the circles shows the population density, ranging from <1000 to >500,000 inhabitants. The collected data indicates that the increase in population density is accompanied by an abandonment of mountainous areas and a marked rural exodus. Both figures show three different situations: an increase in the growth rate in coastal cities and provincial capitals, a moderate growth in the agricultural zone of the Guadalquivir Valley (southwest), and a population decline in natural rural areas.

After having analyzed all the variables collectively, it is clear the apparent increase in the fire risk level in the territory. This can be attributed to an increase in climate hazards resulting from a rising LST trend, as well as to a greater vegetation density on abandoned croplands and forest cover. Furthermore, the current rural exodus and depopulation observed in natural areas, where it has been shown that fires are more frequent, exacerbate this situation and hinder the implementation of an early warning and control system.

### 4.2 Changes in the occurrence of fires and burned surfaces

Below it is reported a descriptive analysis of changes in the occurrence of fires recorded in Andalusia, Spain, between 2001 and 2020.

**Fig. 7:**
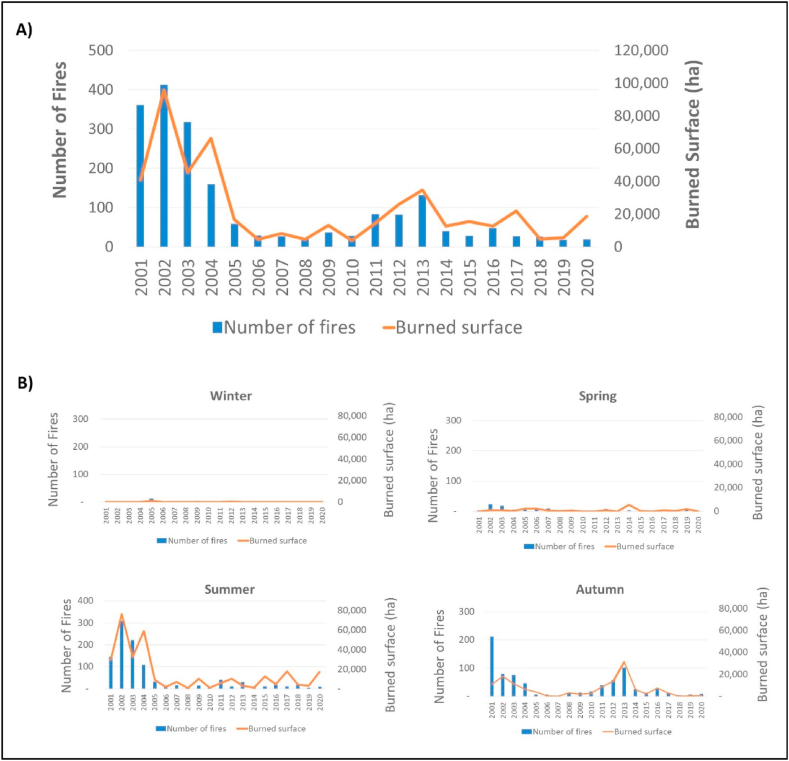
A) Relation between burned surfaces (orange line, right y-axis) and the number of fires (blue columns, left y-axis) in Andalusia between 2001 and 2020. B) Burned surfaces (orange line) and the number of fires (blue columns) by season in Andalusia between 2001 and 2020. (For interpretation of the references to color in this figure legend, the reader is referred to the Web version of this article.)

Fig. 7A displays the relationship between the yearly number of fires and burned hectares in Andalusia, using data obtained from MODIS burned surface analysis. The year 2002 was the most affected with over 90,000 burned hectares and more than 400 fires. However, a decreasing trend in both the number of fires and burned hectares has been observed since then, with a slight increase only over the 2011–2013 period.

Fig. 7B shows the seasonal patterns of fires. During the period of 2001–2004, fires had the most significant impact during the summer. However, from 2011 to 2016, there was a significant increase in the number of fires and burned hectares during the autumn season. This change in trend peaked in 2013, with a rate of 90.5% of burned hectares in autumn. The occurrence of fires during winter and spring was relatively rare.

Table 2 presents the relative annual variation recorded for the period between 2001 and 2020. A decrease has been observed since 2005 in both the number of fires and burned hectares. However, the table indicates that the relative variation in the number of fires is higher than that of burned hectares. This data confirms a more pronounced decrease in this variable and suggests that currently, fires are less frequent but more severe. The increase in burned areas during the spring of 2014 is related to the large fires that occurred in the semiarid zone of southeastern Andalusia at that time. The data obtained through CHIRPS analysis indicates that the spring of 2014 had low precipitation levels and a semestral SPI of −1.5, suggesting the existence of a drought period. The NDVI levels recorded at that time were also lower than usual, which may have facilitated the spread of the fire once it started.

**Table 2:** Relative variations (%) in the number of fires and burned surfaces, and rate of burned surface (%) by season in Andalusia from 2001 to 2020.

**Year**	**Relative variation of Nº of fires (%)**	**Relative variation of burned surface (%)**	**Rate of burned areas in winter (%)**	**Rate of burned surfaces in spring (%)**	**Rate of burned surfaces in summer (%)**	**Rate of burned surfaces in autumn (%)**
2001	0.0%	0.0%	0.0%	0.1%	72.8%	27.1%
2002	14.4%	134.6%	0.0%	1.1%	79.9%	29.0%
2003	−11.9%	11.1%	3.0%	2.1%	72.3%	25.2%
2004	−56.0%	62.3%	0.0%	0.6%	89.6%	9.7%
2005	−83.9%	−59.4%	6.3%	14.2%	55.7%	23.8%
2006	−92.0%	−88.6%	2.5%	46.2%	40.6%	20.7%
2007	−92.5%	−80.4%	2.0%	6.9%	90.1%	1.0%
2008	−94.2%	−89.2%	0.0%	6.9%	17.0%	76.2%
2009	−89.8%	−68.2%	1.2%	5.8%	78.5%	14.5%
2010	−92.2%	−90.8%	0.0%	0.0%	25.2%	74.8%
2011	−77.0%	−64.7%	0.1%	0.1%	41.3%	58.5%
2012	−77.3%	−36.7%	2.0%	3.8%	40.5%	53.8%
2013	−63.4%	−15.1%	0.0%	0.0%	9.5%	90.5%
2014	−88.9%	−69.0%	0.0%	44.3%	7.6%	48.2%
2015	−92.2%	−62.3%	0.0%	1.8%	83.3%	14.9%
2016	−86.7%	−69.4%	1.0%	0.2%	39.3%	59.6%
2017	−92.5%	−46.5%	0.0%	4.0%	82.2%	13.8%
2018	−92.8%	−88.5%	0.0%	8.4%	89.0%	2.5%
2019	−95.0%	−86.4%	0.0%	33.1%	59.7%	7.2%
2020	−94.7%	−54.6%	0.0%	0.0%	95.3%	4.7%

**Fig. 8:**
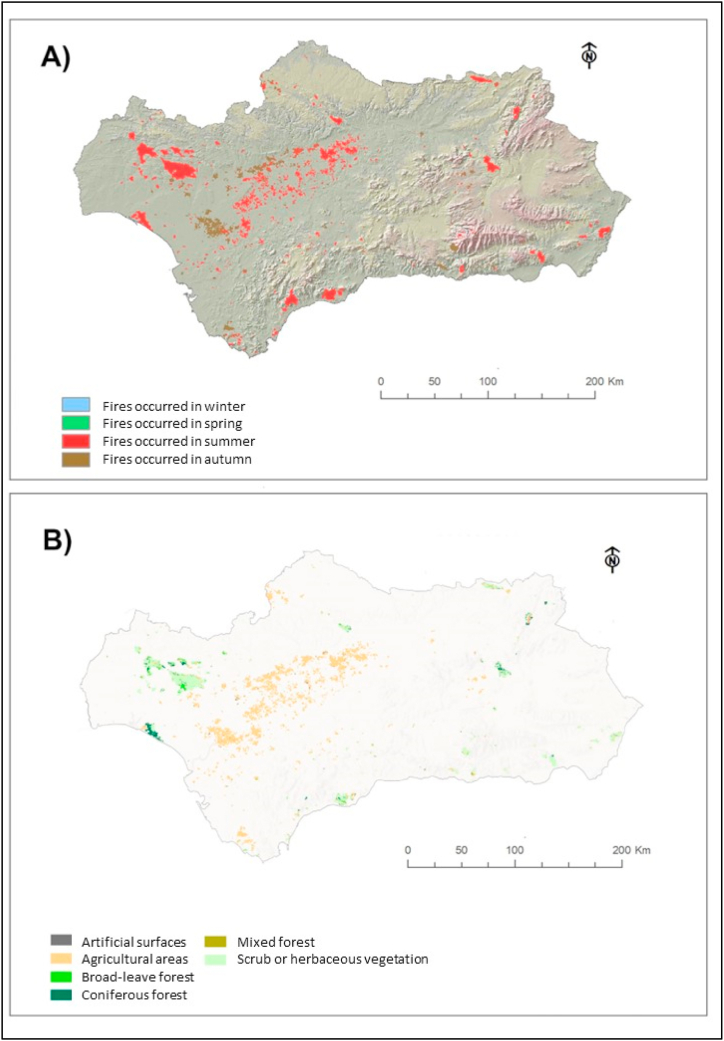
Burned surfaces in Andalusia between 2001 and 2020: A) by season (blue: winter; green: spring; red: summer; brown: autumn); B) by land coverage (yellow: croplands; dark green: forest; light green: scrub). (For interpretation of the references to color in this figure legend, the reader is referred to the Web version of this article.)

Fig. 8A illustrates the spatial distribution of fires over the 2001–2020 period. The color scale indicates the seasonality of fires, mainly occurring in summer and autumn. Fig. 8B shows the relationship between burned surface and land cover type. Generally, fires occur predominantly in agricultural areas (59.9%), followed by scrubland or herbaceous vegetation areas (27.8%), coniferous forests (5%), grasslands (4%), deciduous forests (2.8%), and mixed forests (0.4%). Only 0.09% of fires occur in urban areas and artificial surfaces. Forest areas only experience fires during summer, while shrub and agricultural areas are also affected during autumn. Furthermore, it can be observed that fires in agricultural areas (80%) affect smaller surfaces (<500 ha) than those in natural areas (20%), which affect larger surfaces (5000 and 28,000 ha). Finally, it is noted that large fires of over 500 ha only occur in natural areas.

By examining all the information collectively, two distinct trends in the incidence of fires can be observed. In the early 2000s, there was a substantial number of summer fires, predominantly in agricultural and scrubland areas. From 2011 onwards, the fire season expanded to include autumn months, while the number of fires decreased and the burned hectares mean increased.

### 4.3 Analysis of the meteorological conditions of the 355 studied fires

This section provides a description of the LST, precipitation, and vegetative density variables that were measured in the areas where 355 fires originated between 2001 and 2020. The fires have been categorized into four time periods (2001–2005, 2006–2010, 2011–2015, and 2016–2020) to explore the possible relationship between the territorial-level meteorological variables and the climatic conditions of the 355 areas at the time the fires started.

**Fig. 9:**
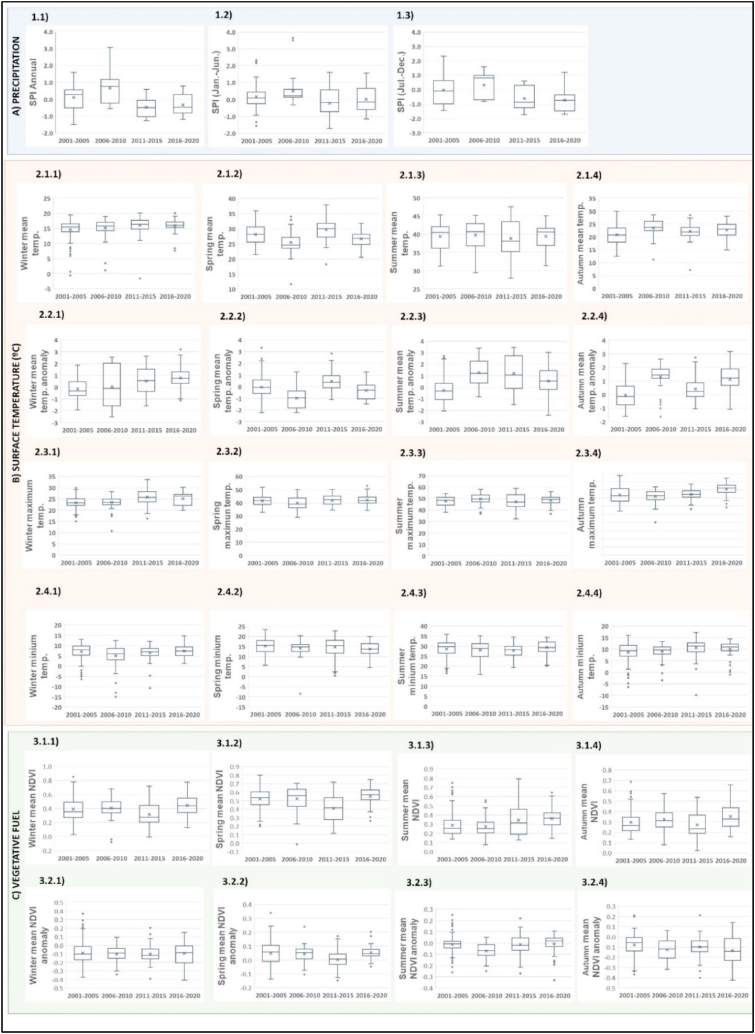
Climate trends for the 335 fires analyzed in Andalusia between 2001 and 2020. A) Precipitation; B) Land Surface Temperature; C) Vegetative density. The code of the plots is indicative of the variable analyzed, of the line in the illustration, and of the plot number.

Fig. 9 illustrates the meteorological and vegetative variables recorded in the 335 analyzed fires, categorized by 5-year periods. The interannual variations represent the meteorological and vegetative changes observed in the different fires that occurred between 2001 and 2020.

Fig. 9A displays the anomalies in precipitation levels based on annual SPI (12 months. Plot 1.1) and semi-annual SPI (6 months, plots 1.2 from January to June and plot 1.3 from July to December). Increasingly negative values indicate that fires occur more frequently in areas that are affected by annual and/or semi-annual droughts during the months leading up to the fire.

Fig. 9B illustrates the changes in LST including the mean, anomalies, maxima, and minima. The LST mean indicates very high values, exceeding 35 °C during the summer in most of the observed fires. The anomalies demonstrate an increase in summer and autumn LST coinciding with the highest occurrence of autumn fires (2011–2015) in the cartography. The maxima and minima LST also demonstrate an increasing trend.

Fig. 9C shows the vegetative density and the available fuel from NDVI values analysis. Values between 0.2 and 0.4 recorded during summer and autumn are indicative of low humidity and easy flammability. During winter and spring, an increase in NDVI is observed due to the phenological growth of plants. Although cartography shows a widespread increase in NDVI at the territorial level, fires continue to occur mainly in shrublands with low vegetative density and low humidity.

If we analyze all the variables together, these are indicative of an increase in the meteorological hazards in the burned surfaces. Nowadays fires are increasingly difficult to control due to the extreme weather conditions that cause their ignition (extreme drought conditions (SPI) coupled with mean LST anomalies) as well as due to extreme conditions they present, during the spread.

**Fig. 10:**
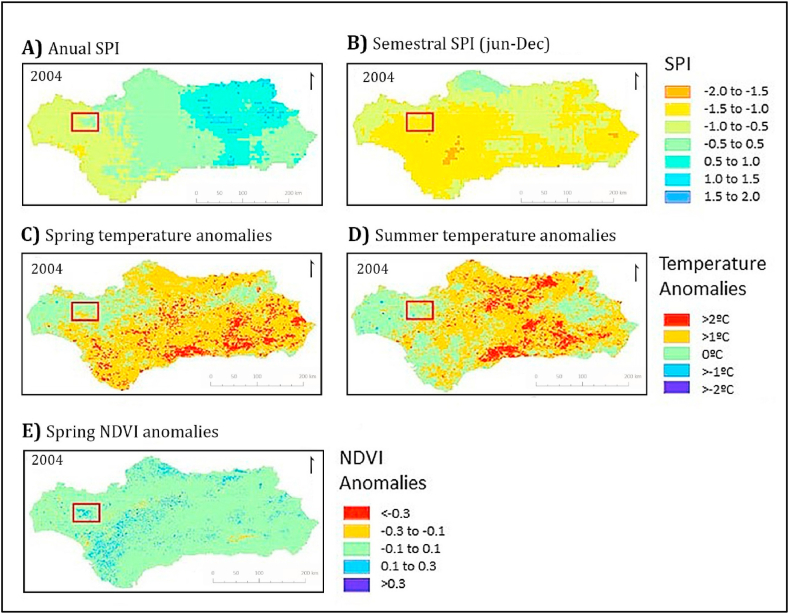
Characteristics of temperature, drought, and vegetative density recorded at the time when the Rio Tinto fire was initiated (July 2004). A) Annual SPI; B) Semestral SPI; C) Spring temperature anomalies; D) Summer temperature anomalies; E) Spring NDVI anomalies. The location of the affected area is highlighted with a red rectangle. (For interpretation of the references to color in this figure legend, the reader is referred to the Web version of this article.)

**Fig. 11:**
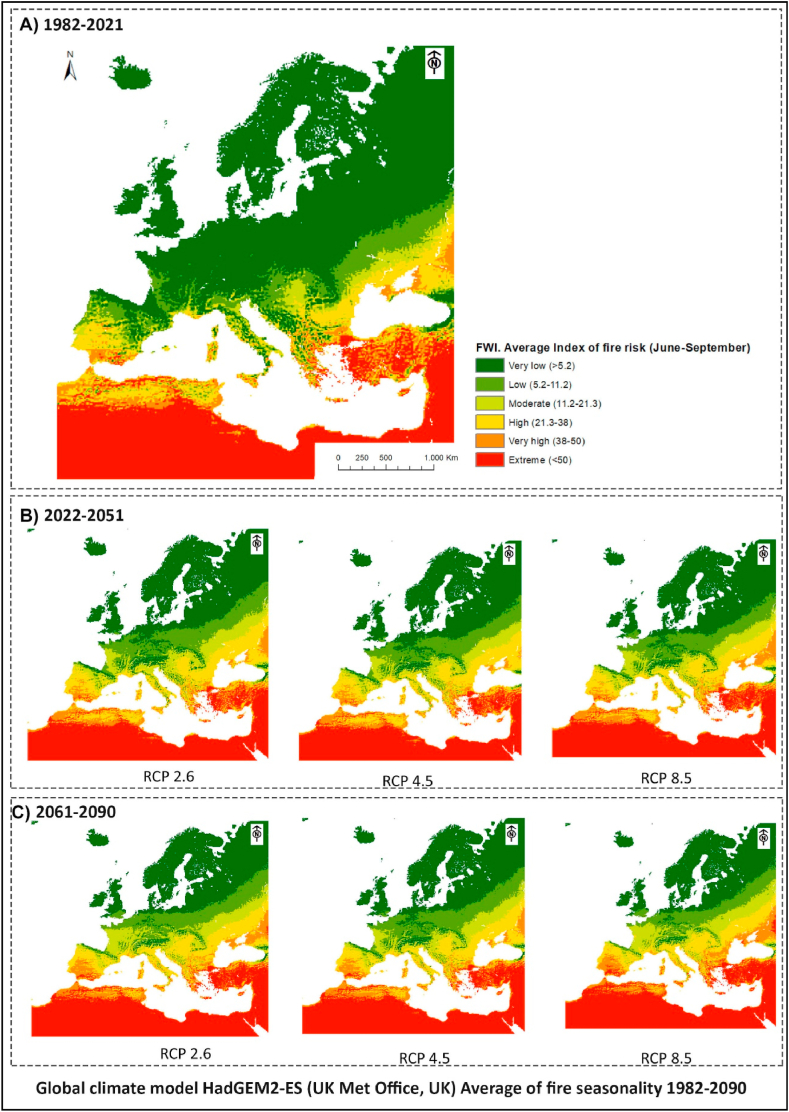
Meteorological risk of fire according to FWI in the Mediterranean basin. A) Present; B) Near future; C) Far future. Maps calculated at different greenhouse gas concentration scenarios. RCP 2.6: reflects a very restrictive path in CO_2_ emission policies. RCP 4.5: shows a trajectory in which CO_2_ concentrations decrease from 2040. RCP8.5 shows a trajectory in which CO_2_ concentrations continue to increase. Data obtained from Copernicus and HadGEM2-ES climate model.

As an example, Fig. 10 shows the Minas del Río Tinto fire, which burned 28.000 ha in 2004. The NDVI levels before this fire presented anomalies of more than 0.3 over the mean NDVI recorded in the area (highlighted with a red box in Fig. 11). The drought identified during the summer of 2004 had an SPI level of −1.5 (Fig. 10B), and the mean spring LST anomaly (Fig. 10C) had a value up to 1 °C, favoring all together the fire development. Then, the increase of forests and shrubs facilitated the rapid expansion of the fire, and the remoteness of the area made difficult its extinction (Fig. 10E).

## 5 Discussion

The territorial cartography developed from satellite resources reveals that since 2001, Andalusia has experienced an expansion of areas registering very high LST, especially during summer and autumn seasons. These conditions are exacerbated by low precipitation and periodic droughts, as indicated by the low levels of the Standardized Precipitation Index (SPI) observed on an annual and semi-annual basis. The region's climate presents a high meteorological hazard during summer, increasing the risk of rapid spread and occurrence of large uncontrollable fires [66].

As a result of these climatic conditions, NDVI levels experience a sharp decrease during summer, increasing flammability due to the lack of humidity in vegetation. Consequently, shrubland areas are the most prone to fires in Andalusia. However, fires that affect this type of vegetation tend to burn smaller surfaces due to their limited fuel capacity and proximity to inhabited areas, allowing for early warning and rapid control. Nevertheless, when shrubland and forested areas coexist in the same region, the risk and probability of a large-scale fire significantly increase. Thus, the large fires that occurred between 2001 and 2020 only affect natural areas that alternate shrubland and forested zones.

The aforementioned meteorological factors have a significant impact on a large part of Mediterranean ecosystems, promoting rapid and uncontrollable fires spread, making it difficult to control them [67]. Fig. 11A shows the mean FWI index calculation during the fire season (June to September) in the Mediterranean basin from meteorological data over the last 40 years. This figure was obtained from statistical analysis of Copernicus cartography and shows the meteorological risk of fires based on LST, precipitation, and wind data. Although the scale does not allow highlighting variations caused by orography, it does show the high risk in the Mediterranean basin due to its climatic characteristics.

In turn, Fig. 11B and C shows the prediction of meteorological risk for a near future scenario (2022–2051) and a distant future scenario (2061–2090). The images were prepared from the HadGEM2-ES climate model and consider the three Representative Concentration Pathways, namely, the moderate RCP.2.6, the medium RCP.4.5, and the high-impact CO2 emission RCP 8.5 scenario. Although current data already indicates the existence of high meteorological risk, simulations show that future climatic conditions could be even more extreme [[60], [61], [62]]. Furthermore, all scenarios show an increase in risk throughout the Mediterranean basin, especially affecting Spain, Greece, Turkey, and North Africa. Regarding precipitation, although an increase in extreme rainfall and a greater likelihood of flooding is expected, forecasts indicate a widespread decrease in annual accumulated rainfall in southern Spain. These results are supported by studies conducted by Ref. [68] and Lorenzo and Alvarez [40]. In this context, it is highly likely that climate change will encourage the occurrence of large fires in the future [69]. Therefore, the findings of this research coincide with those of other studies carried out in Mediterranean climate zones, which have related droughts and other effects of climate change to the large fires that have ravaged Mediterranean climate areas in recent times [70].

In the case of Andalusia, the increase in meteorological risk has been accompanied by a change in fire regimes. The analysis carried out identifies two distinct trends between 2001 and 2020. The first trend, which extends until 2013, reflects the occurrence of numerous fires with little burned area located mainly in agricultural areas. This trend is first related to the agricultural use of land and later to the growth of shrubs in abandoned crops. The increase in the number of fires and the change in seasonality recorded between 2010 and 2013 is probably related to the control of small fires that occurred during the summer, which led to an increase in available vegetal fuel in the autumn. Since 2014, a second trend has been observed, which shows a smaller number of fires capable of burning more hectares. This second trend can be attributed to the beginning of more dangerous fire regimes driven by meteorological factors and located in natural areas that are currently affected by depopulation and rural exodus.

From a social point of view, Mediterranean cultural landscapes have historically been characterized by ecosystems in which human settlements and natural areas intermingle [19], as well as by the traditional use of fire in agricultural, livestock, and forestry activities [[71], [72], [73]]. These territories present common traditions and ways of exploiting the environment adapted to the management of territories with a high meteorological fire risk. In this context, the loss of local traditions not only implies a loss of identity but also increases the levels of fire risk.

Between 2001 and 2014, fires in Andalusia occurred more frequently in agricultural areas. This coincided with a significant rural exodus in the Guadalquivir agricultural valley due to the 2008 economic crisis [[74], [75], [76]]. Currently, there is a trend of larger fires in natural areas, which corresponds to depopulation of mountain villages near these areas.

The new territorial dynamics developed throughout the 21st century in many of the socioeconomic landscapes of the Mediterranean basin increase the fire risk [43,77,78]. The growth of cities has generally led to abandonment of rural and cultivated areas, increasing the presence of shrubs. Additionally, local communities' knowledge of how to use fire properly has also weakened, favoring the development of fires due to negligence [10]. This situation is a common feature of most Mediterranean countries where there is no policy for disseminating knowledge of traditional fire use practices that include how to use and control the fire [10]. According to studies developed by other authors [[79], [80], [81]], this could be one of the main causes of agricultural fires in regions where the proportion of agricultural land remains high, such as southern Spain, Italy, or Slovakia.

In Andalusia, from 2015 onwards, there has been an increase in the occurrence of large fires in natural areas. In these cases, high LST and low precipitation have favored the ignition of the scrub and the subsequent burning of the trees. The high proportion of combustible material available in summer increases the intensity of the fire and makes it difficult to extinguish. Additionally, since forests are natural areas far from populated centers, the alert system works slowly in this type of fire.

The increase in thermal anomalies during autumn extends the high-risk fire season and makes the territory more vulnerable during months where fires traditionally did not occur. This trend is a characteristic feature of the Mediterranean basin due to climate change. The recent change in the seasonality of fires may also be due to firefighting policies and careful preparation, which increase surveillance during the summer and delay the occurrence of fires until autumn when protection services are instead reduced.

In agricultural areas, where most fires occur during autumn, reviving traditional knowledge associated with fire culture and management can be useful in preventing the occurrence of fires and in increasing the resilience of the environment.

In rural areas facing depopulation, traditional agricultural and pastoral practices are in danger of disappearing. These practices helped eliminating vegetative fuels and reducing the risk of fires. To increase the resilience of these areas against high-risk scenarios caused by climate change, it is crucial to maintain and recover landscapes that alternate between agricultural fields and pastures. It is also important to manage and use forest biomass and promote traditional agroforestry systems [43,47,78,82].

Besides, peri-urban areas and scattered housing in semi-natural zones have experienced an increase in fire risk levels in recent years. Most fires in Andalusia are caused by humans, which means that the probability of ignition is higher in these areas than in uninhabited natural areas. Furthermore, the presence of roads and the traffic of summer vacationers and tourists, particularly during the summer months, further increases the risk levels. It is essential to inform and train local populations and visitors about the existing dangers, evacuation routes, and preventive and response measures that can help minimizing risk.

From this theoretical framework, the Common Agricultural Policy (CAP) and laws for rural development can help reviving rural and semi-natural areas economically and minimizing the risk of fire. To achieve this, specific sections related to cultural ecosystem resources need to be included. These sections would promote the development of activities that preserve the use and management of fire culture in agriculture, livestock, forests, history, and heritage of Mediterranean cultural landscapes.

## 6 Conclusions

This study describes the variables of fire risk and analyzes the fires registered in Andalusia from 2001 to 2020 with the aim of identifying the main factors that influence the creation of high-risk fire scenarios. To this end, LST, precipitation, and vegetation data are obtained from satellite resources, as well as data on population distribution, which are discussed. One of the main limitations of this study is that no satellite images are available for burned areas prior to the year 2001. Therefore, it is necessary to complement the analysis carried out with past remote sensing data with data from ground-based meteorological stations.

The obtained cartography indicates a growing trend in mean LST and vegetative density at the territorial level. This increase in risk associated with climate change has also been identified throughout the southern area of the Mediterranean basin.

The analysis of the 335 fires shows that although there has been a decrease in the number of fires and burned surface hectares in Andalusia in recent years, there has also been an increase in the risk of intense large fires, that are more difficult to control due to the new meteorological characteristics.

The changes observed in the historical fire regimes in Andalusia coincide with moments of rural exodus and depopulation, which must be understood as a result of the joint action of meteorological and demographic factors. In this context, the revitalization of rural territories, cultural landscapes, and intangible fire culture can create alternatives for minimizing the risk of large fires. Last but not least, to better understand how social and cultural factors influence the creation of high-risk fire environments, it is important to analyze the geospatial and territorial dimension of qualitative social variables. Therefore, it is recommended that future research works focus on these aspects to obtain a more complete understanding of fire risk scenarios. It is urgent to improve sustainable management due to the exacerbation of hazard caused by ongoing climate change, which is showing its first signs of appearance.

## Author contribution statement

Conceived and designed the experiments: Mónica Moreno, Chiara Bertolin, Pilar Ortiz.

Performed the experiments: Mónica Moreno, Daniel Arlanzón.

Analyzed and interpreted the data: Mónica Moreno, Chiara Bertolin, Rocío Ortiz, Pilar Ortiz.

Contributed reagents, materials, analysis tools or data: Mónica Moreno, Daniel Arlanzón.

Wrote the paper: Mónica Moreno, Chiara Bertolin, Rocío Ortiz, Pilar Ortiz.

## 7 Data availability statement

Data will be made available on request.

## Declaration of competing interest

The authors declare the following financial interests/personal relationships which may be considered as potential competing interests:

This study has been carried out thanks to FENIX (PID2019-107257RB-I00) financed by MCIN/AEI//10.13039/501100011033/FEDER “A way to build Europe”; RESILIENT-TOURISM (PYC20 RE034 UPO) project of Consejería de Transformación Económica, Industria, Conocimiento y Universidades, Junta de Andalucia; Diagnosis and Cataloging of Andalusian Architectural Heritage through Risk and Vulnerability Analysis (UPO 20.01) project of Consejeria de fomento, infraestructura y ordenación del territorio, Junta de Andalucia; and the research teams TEP-199 (Patrimonio, Medioambiente y Tecnología) and Sanit-ARTE laboratory.

Mrs. Moreno is grateful to the State Program for the Promotion of Talent and its Employability of the 10.13039/501100004837Ministry of Science and Innovation of Spain for his technical fellowship (PTA2019-016882) funded by MCIN/AEI/10.13039/501100011033.
